# Supplementary material for: A Global Screen for Assembly State Changes of the Mitotic Proteome by SEC-SWATH-MS
Source: Cell Syst. 2020 Feb 26;10(2):133–155.e6. doi: 10.1016/j.cels.2020.01.001 (PMC7042714; doi:10.1016/j.cels.2020.01.001)

Q96JB5 | CK5P3_HUMAN | CDK5RAP3 IC53 LZAP MSTP016 OK/SW−cl.114 PP1553
Monomer MW [kDa]: 56.921 Monomer expected elution fraction: 45

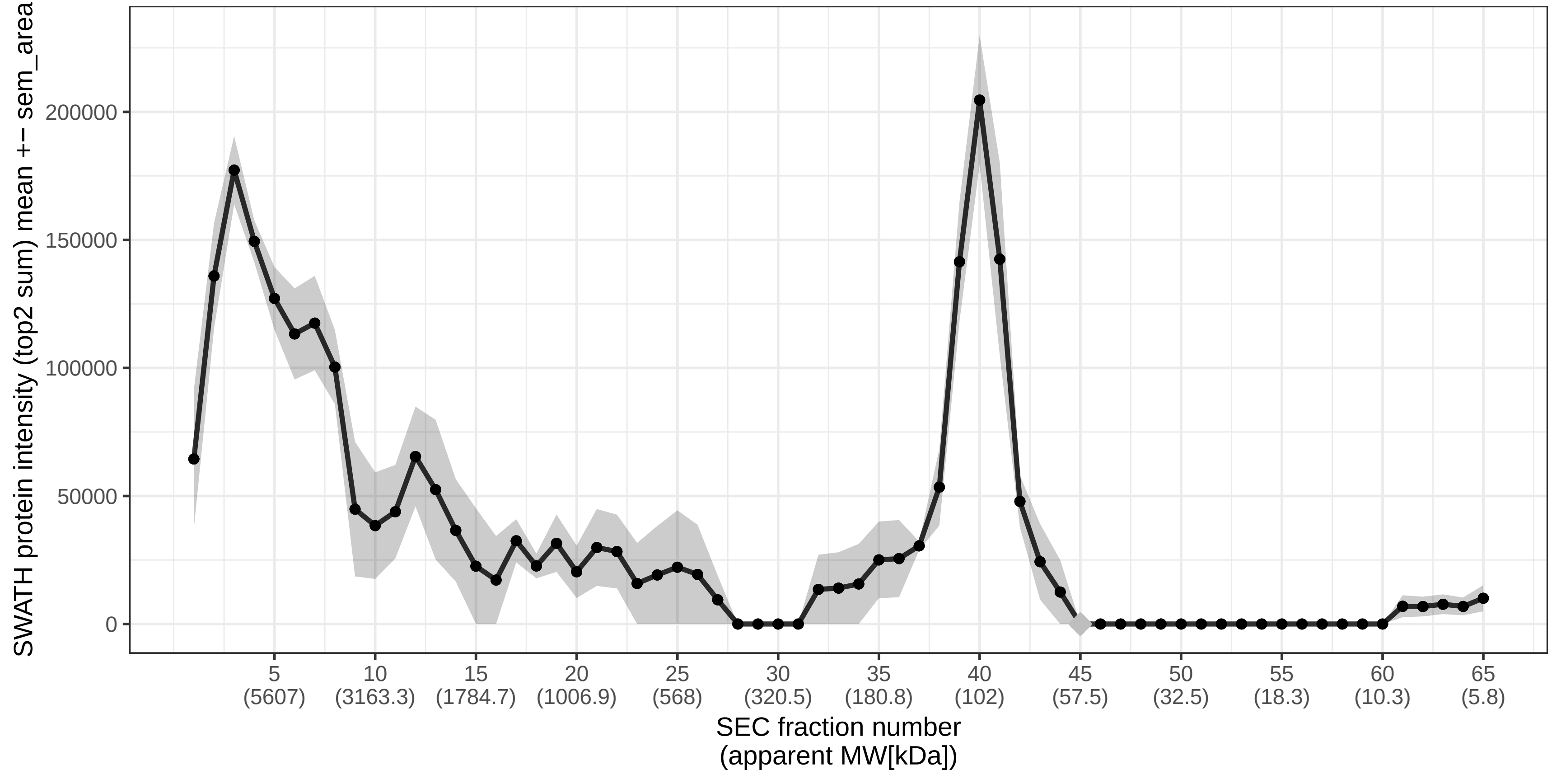

Supplement: Data S1. SEC-SWATH-MS Protein Chromatograms, Related to Figure 1 [file mmc6.zip › SECchrom_Q96JB5_CK5P3_HUMAN_CDK5RAP3_IC53_LZAP_MSTP016_OK_S.pdf]
